# Adolescent endometriosis: clinical insights and imaging considerations

**DOI:** 10.1007/s00261-025-04870-7

**Published:** 2025-03-21

**Authors:** Brooke S. Lampl, Cara R. King, Marjan Attaran, Myra K. Feldman

**Affiliations:** 1 Imaging Institute, Cleveland Clinic, Cleveland, United States; 2 Ob/Gyn and Women’s Health Institute, Cleveland Clinic, Cleveland, United States

**Keywords:** Endometriosis, Adolescent endometriosis, MRI endometriosis, US endometriosis, Deep endometriosis

## Abstract

Endometriosis is a gynecologic disorder characterized by endometrial-like tissue in ectopic locations, outside of the uterine cavity. In the adolescent population, the diagnosis of endometriosis is often overlooked as a cause of pelvic pain because of limited awareness by both patients and providers and a tendency to undervalue the complaints of pain by both families and caregivers. Although historically diagnosed by laparoscopy, there has been a shift in recent years towards imaging diagnosis. Transvaginal ultrasound is considered the first-line imaging technique for endometriosis in the adult population; however, this minimally invasive procedure is commonly avoided in adolescents, with MRI often used instead. Here, we discuss the clinical presentation and imaging considerations and treatment options involved in adolescents with suspected endometriosis.

**Graphical abstract:**

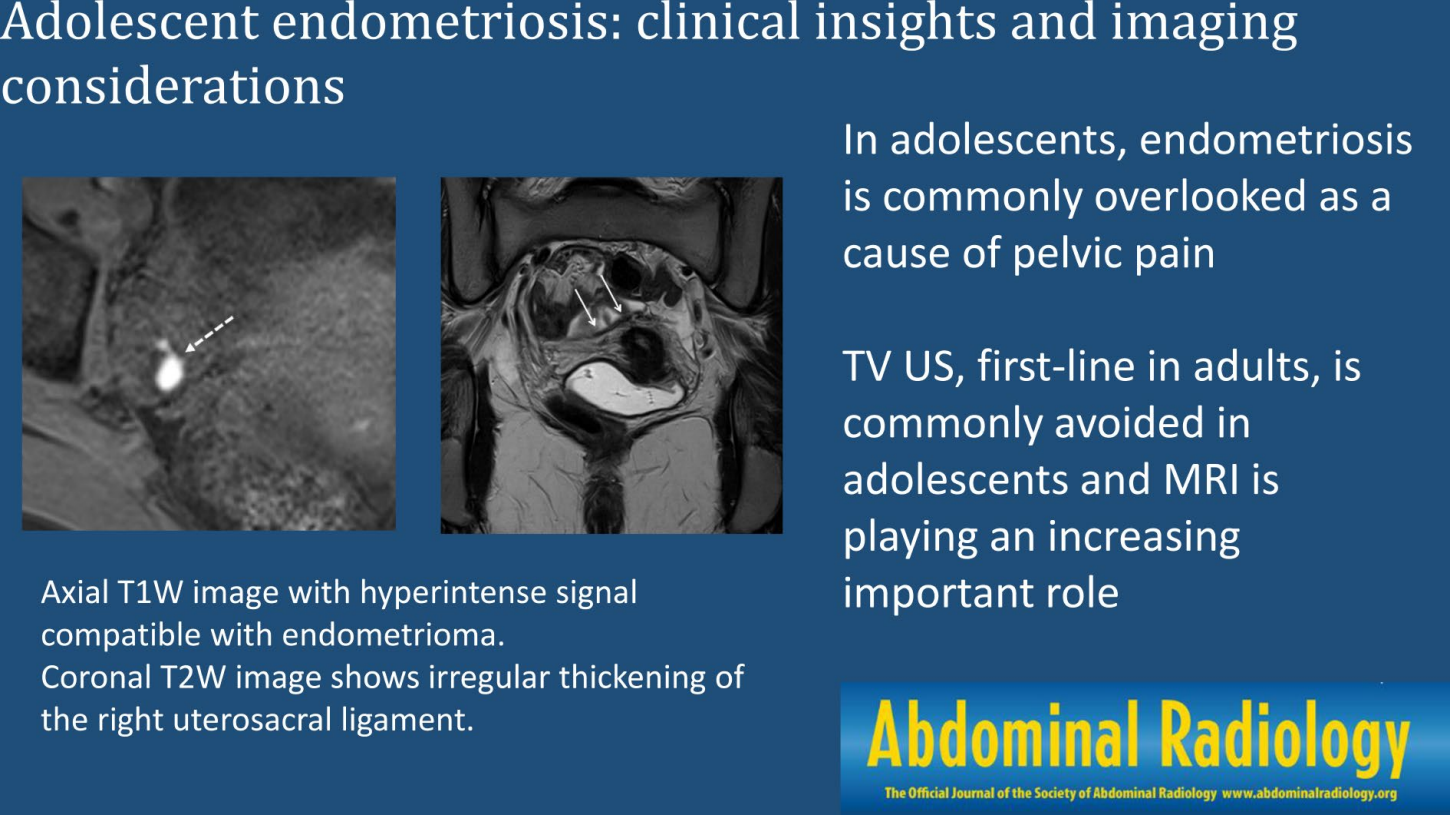

## Introduction

Endometriosis is a gynecologic disorder in which endometrial-like tissue is found outside the uterine lining, often associated with inflammation [1]. The clinical presentation is variable, with pelvic pain comprising the most frequent symptom across all age groups. In the adolescent population, endometriosis is often overlooked as a cause of pelvic pain because of limited awareness by caregivers as well as a confusing clinical picture, where patients may present with cyclical and acyclical pain. This combination of factors may lead to a delay in diagnosis, which can have considerable clinical implications, such as persistent pain, central sensitization, and potential infertility. Adolescents, generally considered between the ages of 10 and 19 years, are a unique patient cohort in that they have started puberty but are not yet adults. The adolescent population may feel uncomfortable discussing symptoms or may consider their symptoms normal. Additionally, adolescents are more likely than previously thought to develop deep endometriosis, a severe form of endometriosis involving tissues below the peritoneum.

Although the diagnosis of endometriosis in adults is often confirmed by laparoscopy, there has been increased emphasis on diagnosis and surgical planning via imaging in recent years [2]. In the adult population, transvaginal ultrasound is considered the first-line imaging technique for clinically suspected endometriosis, with magnetic resonance imaging (MRI) reserved as a second-line imaging study to confirm the diagnosis and for treatment planning [3]. In contrast, in the adolescent population, transabdominal ultrasound is the first-line imaging technique for pelvic pain. Many adolescents who are not sexually active may feel uncomfortable undergoing transvaginal ultrasound, although it is not contraindicated in the nonsexually active patient. A transabdominal ultrasound can be helpful for the identification of ovarian endometriomas but has a limited role in the evaluation of deep pelvic endometriosis. Therefore, in these patients, MRI is playing an increasingly important role in diagnosis [4, 5]. This paper discusses the nuances associated with the clinical and imaging diagnosis of suspected endometriosis in adolescents.

## Presentation

Endometriosis is a chronic condition in which ectopic endometrial glands and stroma are found outside of the uterus. It is the most common cause of secondary dysmenorrhea in adolescents. In a review by Janssen et al. [6], 70% of adolescents undergoing laparoscopy for dysmenorrhea unresponsive to nonsteroidal anti-inflammatory drugs and suppressive hormone therapy were diagnosed with endometriosis.

Endometriosis can present with a broad spectrum of symptoms. Classically, the adult patient experiences severe dysmenorrhea associated with luteal phase symptoms of pelvic pain. However, patients can have symptoms ranging from chronic daily pain to dyspareunia, cyclic bowel and bladder symptoms, and infertility. In adolescents, noncyclic pain or pain between periods is a common complaint. In one retrospective study, more than 90% of adolescents with endometriosis complained of acyclic pain, and 62% complained of both acyclic and cyclic pain [7]. Other symptoms may include irregular heavy periods, dyspareunia, and nausea. Adolescents and young adults less than 24 years of age are far more likely to report higher pain scores than older patients [8].

### Diagnostic delay

Diagnostic delay is common in adolescents and is driven by factors involving patients and clinicians. Of note, adolescents wait three times longer than adults to seek medical care [9]. Many factors lead to adolescents normalizing the degree of pain they are experiencing. Young adolescents often experience embarrassment and will not commonly share information about their periods with family or friends. They do not realize that the degree of pain they are experiencing is abnormal and are led to believe that menstrual cramps are an expected part of the menstrual cycle through societal norms. When clinicians obtain a medical history from an adolescent, it is important to determine the extent to which this pain is interfering with school and extracurricular activities, as absenteeism is noted to be much higher in adolescents with endometriosis [8]. Their quality of life is dramatically impacted, leading to poor socialization and concern for isolation at a particularly vulnerable time of their lives. Adolescents with endometriosis are more likely to report mental health issues such as anxiety and depression, the use of pain medications, and the avoidance of exercise [10].

Clinicians are less likely to consider a diagnosis of endometriosis in adolescents than in adults. The difference in clinical presentation and lack of specific findings on pelvic examination and ultrasound often mislead the clinician. The pelvic examination in adolescents with endometriosis is often relatively benign compared to the pelvic examination in adults with endometriosis, which is more likely to reveal a pelvic mass or nodularity. Additionally, the common site of disease, the posterior cul-de-sac and uterosacral ligaments, can be challenging to evaluate on physical exam, particularly in patients who have not been sexually active. Thus, clinicians are less likely to order pelvic ultrasound examinations for adolescents and are more reluctant to recommend diagnostic laparoscopy, which could be perceived as a relatively aggressive diagnostic measure in such young individuals. However, laparoscopy is paramount for diagnosis in this population, as most endometriosis lesions in these patients are superficial and not likely to be noted via imaging studies. In addition, endometriosis lesions in adolescents often do not have the classic powder-burn, black or brown endometrial tissue, on direct visualization by laparoscopy and therefore can easily be missed by Gynecologic surgeons. These atypical lesions, often presenting as clear, white, or small hemorrhagic lesions, produce high levels of prostaglandins and substantial pain [8, 11].

## Diagnostic imaging considerations

The need for imaging should be determined by both history and physical examination, as imaging is not routinely needed in a patient with mild dysmenorrhea. Evaluation of the adolescent with symptoms of endometriosis should begin with a detailed history regarding the type and location of pain, cyclic association, a positive family history of endometriosis, a history of obstructive genital malformations, early menarche, short menstrual cycles, and the use of contraceptives to treat severe dysmenorrhea, as well as inciting factors [11, 12].

Standard imaging evaluation for the adolescent patient with concern for endometriosis should begin with a transabdominal pelvic ultrasound examination, which is considered first-line imaging, particularly in young patients who are not sexually active. Although transabdominal ultrasound is limited in its evaluation of endometriosis, it may exclude other causes of pain such as adnexal masses or congenital anomalies [8]. Additionally, transabdominal ultrasound may identify some forms of endometriosis, including endometriomas; however, these are a less common manifestation of endometriosis in the adolescent population.

After a transabdominal ultrasound examination has been performed, one may consider performing a transvaginal ultrasound (TVUS). Although this technique is superior to transabdominal ultrasound in its evaluation of the female pelvis and specifically in identifying endometriosis, it is a sensitive procedure that may be considered in the adolescent after considerable thought and care. Transvaginal ultrasound is less commonly used in adolescent patients who are not sexually active and may cause anxiety and discomfort even in those who are sexually active; however, as a gold-standard technique, it can still be offered to women of reproductive age after a discussion of risks and benefits. In some circumstances, transrectal or transperineal ultrasound may be considered in nonsexually active patients [13]. Table 1 outlines standard transabdominal and transvaginal ultrasound protocols for the evaluation of endometriosis.

**Table 1:** Transabdominal and transvaginal ultrasound protocols for the evaluation of endometriosis

Pelvic structure	Planes	Comment
Uterus	Longitudinal and Transverse	
Endometrium	Longitudinal	With Cine
Ovaries and adnexa	Longitudinal and Transverse	With or without Doppler
Bladder	Longitudinal and Transverse	
Cul de sac and posterior compartment*	Longitudinal and Transverse	Cine sliding sign

*Transvaginal protocol only

Given the limited value of transabdominal ultrasound in the evaluation of deep endometriosis and the potential concerns associated with transvaginal ultrasound, MRI is a helpful tool in the evaluation of adolescents with dysmenorrhea. With appropriate preparation, most adolescents are able to tolerate MRI and do not require anesthesia. Furthermore, adolescent patients with endometriosis are more likely to develop deep endometriosis than previously thought, which is better evaluated with MRI than Ultrasound. MRI is noninvasive and provides excellent soft tissue detail without radiation [14]. MRI of the pelvis may be performed without or with intravenous (IV) contrast in adolescents; adolescent patients can generally tolerate the placement of an IV catheter. Vaginal contrast (aqueous gel) has been shown to improve the detection of endometriosis, particularly that of deep endometriosis, but the use of this contrast may be considered uncomfortable or invasive by adolescent patients [5]. Patients are asked to self-insert the gel before imaging, but if they refuse or are unable to insert the contrast, MRI may still be performed [5]. Fasting for at least 4 h prior to imaging and/or intestinal peristalsis agents may be helpful to decrease bowel motion artifact [15]. Table 2 summarizes the full MRI protocol commonly used to evaluate endometriosis. An abbreviated protocol, utilizing volumetric T2-W 3D SPACE and T1-W Vibe Dixon axial pre-contrast fat saturated images, has also been suggested in the evaluation of endometriosis as the disease often occurs in a predictable pattern. The abbreviated protocol may be useful in younger patients or those with anxiety and can help decrease overall throughput time [15].

**Table 2:** MRI protocol for the evaluation of endometriosis

Sequence	Plane	slice thickness (mm)	comment
T2WI TSE	Axial	3–5	Small FOV for female pelvis
	Coronal and/or sagittal	4	Large FOV
T1WI in-phase and out-of-phase GRE or Dixon	Axial	2–3	Large FOV
T1WI with fat suppression non-contrast	Axial	4	Small FOV for female pelvis
	Sagittal	3–4	Large FOV
T1WI with IV contrast	Axial	3	Small FOV, Subtraction images
	Sagittal	3–4	Large FOV, Subtraction images
Diffusion-weighted Imaging	Axial	4–5	b values = 0, 50, 1000 s/mm2
T1WI pre-three-dimensional fast spoiled GRE with fat suppression	Coronal Sagittal	1	Optional of upper abdomen
T2WI FRFSE with fat saturation	Coronal Sagittal	5	Optional of upper abdomen
T2WI fast-spin echo-planar fast spin echo	Coronal		Large FOV to include kidneys
T2WI fast spin echo with fat saturation	Axial		Large FOV

Adapted from VanBuren W, Feldman M, Shenoy-Bhangle AS, Sakala MD, Young S, Chamie LP, Giudice L, Hindman NM, Tong A, Rabban JT, Yano M, Kilcoyne A, Dave HD, Poder L, Kho RM, Burnett TL, Khan Z, King C, Shen L, Colak C, Burk KS, Andrieu PIC, Franco IVP, Glanc P, Kielar AZ, Taffel MT, Kania LM, Bonde A, Pectasides M, Arif-Tiwari H, Laifer-Narin S, Nicola R, Jha P (2024) Radiology State of the art Review: Endometriosis Imaging Interpretation and Reporting. Radiology 312:e233482. 10.1148/radiol.233482

Ultimately, if imaging is negative and patients are refractory to clinical treatment, the reference standard for the diagnosis of endometriosis in adolescent patients remains laparoscopy, with diagnosis and treatment occurring at the same time. Imaging, however, assists in earlier diagnosis and treatment of adolescent patients, who traditionally experience delays in diagnosis as well as aids in preoperative management which may require a multidisciplinary team in more severe cases.

### Imaging findings

It is known that endometriosis phenotypes evolve throughout a patient’s lifetime. Although adolescents may present with superficial endometriosis, deep endometriosis, or ovarian endometriomas, the proportion of superficial disease is greater in adolescent patients, with the proportion of deep endometriosis and endometriomas increasing after age 24 [16]. An MRI study of endometriosis in patients aged up to 20 years found that the prevalence of endometriosis on MRI increased linearly with age [17]. In this study, retrocervical lesions (uterosacral ligaments and posterior vagina) were seen in 4.7% of patients aged younger than 15 years, in 30% of patients aged 15 to 18 years, and in up to 65% of patients aged 18 to 20 years. Rectosigmoid lesions were not seen in any patients younger than 15 years, and no bladder or ureter lesions were identified in their cohort. Ovarian endometriomas were observed in 20.7% of the patients in this study [17].

Superficial endometriosis is defined as endometriosis along the surface of the peritoneum or organs without subperitoneal extension. These lesions are reliably identified by laparoscopy but not routinely identified on imaging studies. There is evidence that superficial endometriosis can be identified on transvaginal ultrasound examinations as hypoechogenic nodules measuring less than 5 mm in characteristic locations such as along the uterosacral ligaments, but detection of such nodules is operator dependent, and identification of superficial endometriosis by transabdominal ultrasound has not been described. With MRI, superficial disease can be identified as hyperintense foci in characteristic locations on T1-weighted (T1W) images [18].

**Fig. 1:**
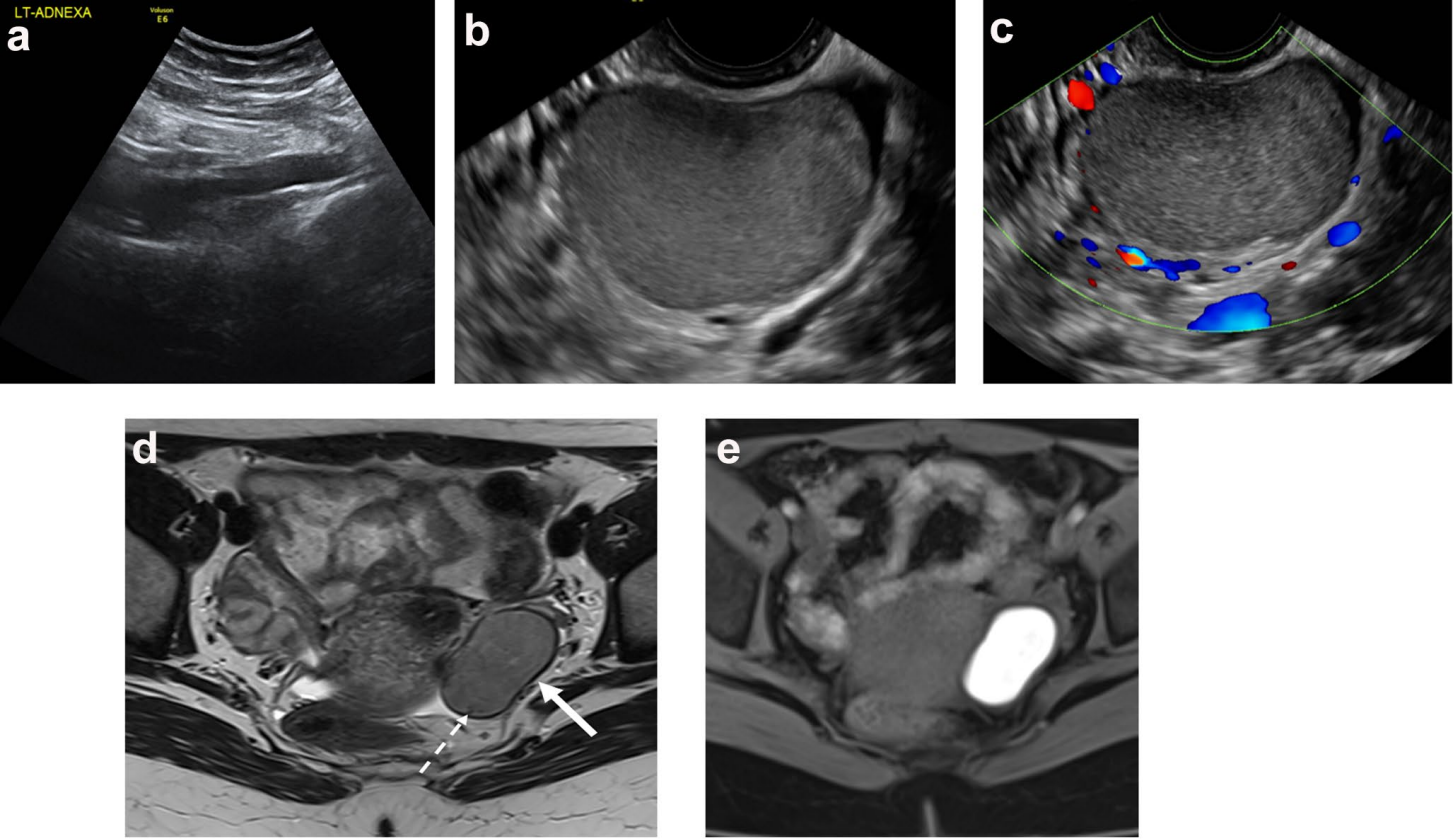
18 year-old woman with left-sided pelvic pain. **a** The ovaries were not identified by transabdominal ultrasound technique. **b**,** c** The patient was amenable to transvaginal ultrasound which showed a unilocular left ovarian cystic lesion with homogeneous low-level internal echoes and no internal vascular flow with color Doppler. **d** The lesion shows characteristic T2 shading on T2W MR image (solid white arrow) with a peripheral dark spot (dashed white arrow). **e** The lesion shows characteristic homogeneous hyperintense signal on T1W image with fat suppression. Patient underwent laparoscopy 2 months later with cystectomy of the endometrioma. Superficial endometriosis was noted in the posterior cul-de-sac

**Fig. 2:**
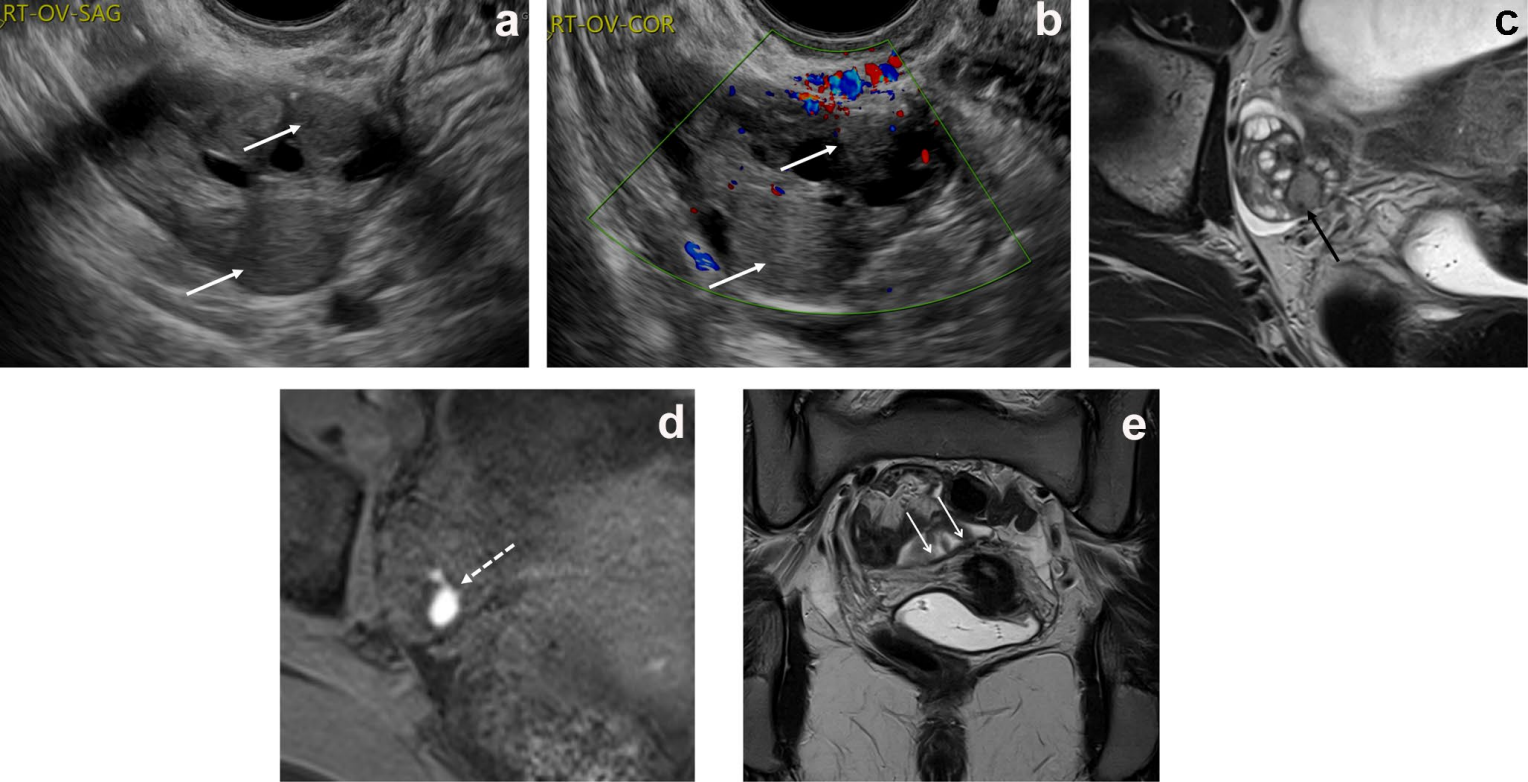
Images from a 19-year-old patient with chronic pelvic pain, dyspareunia, and a family history of endometriosis. **a** Transvaginal ultrasound image through the right ovary shows 2 small cystic structures with low-level internal echoes characteristic of endometriomas (white arrows with closed arrowheads). **b** Image through the right ovary with color Doppler shows no vascular flow within the lesions (white arrows with closed arrowheads). Pelvic MRI was performed 9 months later. **c** T2W axial image shows one of the 2 endometriomas with T2 shading and a dark spot sign (black arrow). **d** The lesion shows characteristic homogeneous hyperintense signal on the corresponding axial T1W image (dashed arrow). **e** Coronal T2W image through the torus uterinus/uterosacral ligaments shows asymmetric, irregular thickening of the right uterosacral ligament (white arrows with open arrowheads). Oral contraception was initiated after imaging

Ovarian endometriomas are ovarian cystic lesions that contain blood products of variable age. When present, these endometriomas can be identified by characteristic imaging features on ultrasound examinations and MRI as described by the American College of Radiology (ACR) Ovarian Reporting and Diagnosis System (O-RADS). On ultrasound examinations, lesions can be unilocular or may contain a few internal septa. Endometrioma cyst fluid has characteristic homogeneous, low-level echoes, sometimes referred to as ground-glass echoes. The inner walls of the cyst should be smooth, and punctate echogenic foci may be observed within the wall. There should be no vascular flow on Doppler imaging within an endometrioma (Figs. 1a-c and 2a and b) [19]. On MRI, endometriotic cyst contents are homogeneously hyperintense on T1W images. Endometriotic cyst fluid is hypointense or intermediate on T2-weighted (T2W) images, often referred to as “T2 shading,” which can appear as homogeneous or graduated. On T2W images, endometriomas may have hypointense nodules or linear mural foci that do not enhance. Endometriomas show variable diffusion signal. Septa may be present but should not enhance (Figs. 1e and 2c and d) [20].

Deep endometriosis is typically multifocal but tends to occur in characteristic locations in the pelvis. The uterosacral ligaments, which are paired uterine suspensory ligaments that extend from the torus uterinus (posterior cervix) posteriorly to the sacrum, are the most common location of deep endometriosis during adolescence (Fig. 2e).

Deep endometriosis lesions are not reliably seen on transabdominal ultrasound studies, but the diagnosis of deep endometriosis by transvaginal ultrasound has been well described [21, 22] and typically focuses on identification of direct or indirect imaging findings of endometriosis. Direct imaging findings are sonographic observations that correspond with ectopic endometrial-like tissue in characteristic locations for endometriosis such as the uterosacral ligaments, uterine serosa, rectosigmoid region, bladder, and vagina. These lesions are typically hypoechoic nodules or areas of hypoechoic thickening with smooth or irregular/spiculated borders. Hyperechoic or cystic foci may be observed within the lesions. Indirect findings are sonographic observations that are a result of deep endometriosis implants but do not correspond with the lesions directly. Indirect observations may include fixed uterine retroversion, abnormal position of the ovaries, and tethering of bowel loops to the uterus [2]. Sliding maneuvers performed along the posterior uterine wall and rectum, along the anterior uterine wall and bladder and at the level of both ovaries can also provide indirect evidence of endometriosis when abnormal sliding between structures is observed [2].

**Fig. 3:**
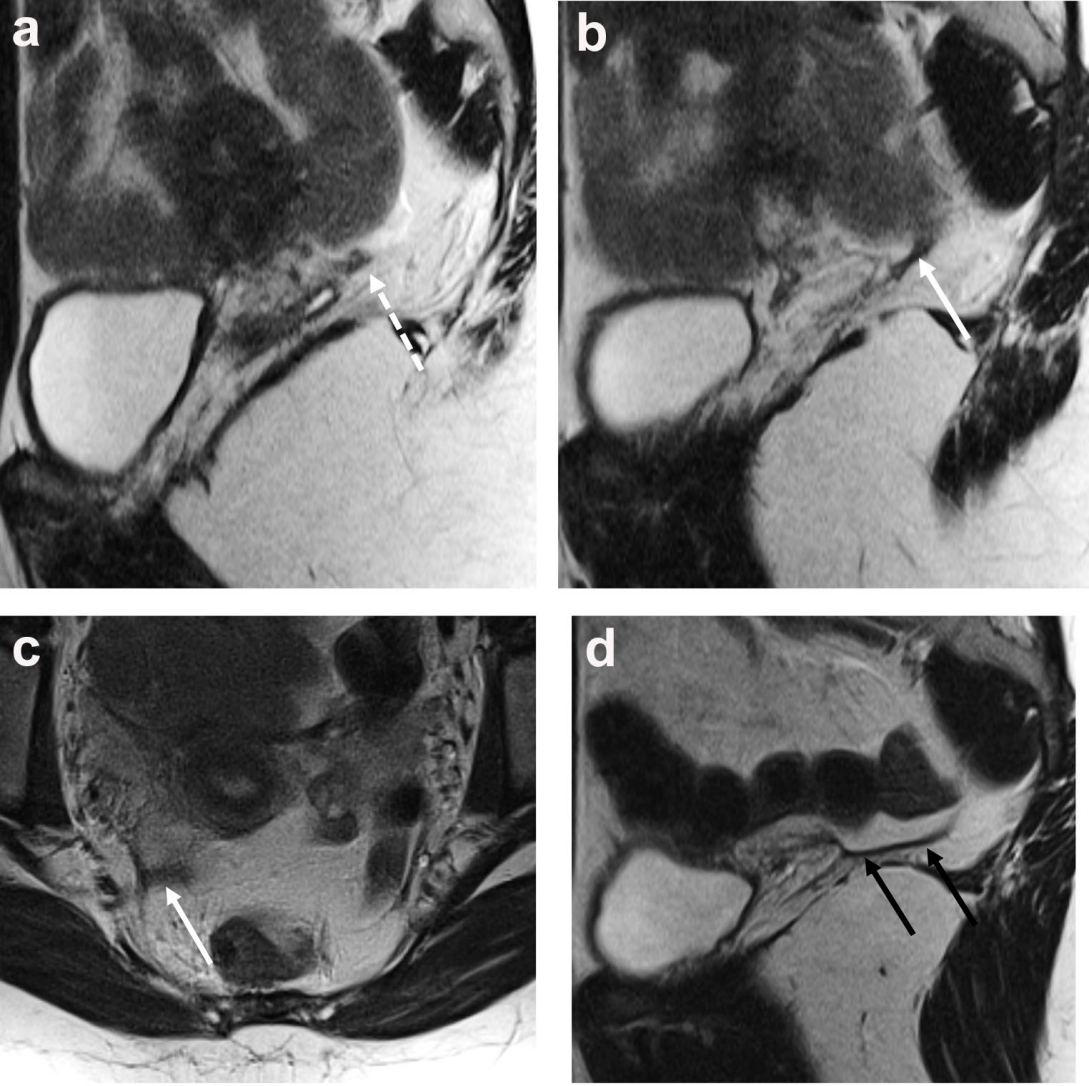
Images from a 15-year-old patient with chronic pelvic pain, dysmenorrhea, and menorrhagia. The patient’s mother had a history of endometriosis. **a**,** b** Sagittal T2W images show a hypointense nodule along the proximal right uterosacral ligament (white dashed arrow) and asymmetric, irregular thickening along the length of the right uterosacral ligament (solid white arrow). **c** Axial T2W image shows that the right uterosacral ligament is also thickened in the axial plane (solid white arrow). **d** Sagittal image through the left uterosacral ligament for comparison shows the ligament to be smooth with no nodules (black arrows). No other sites of deep endometriosis were identified. The ovaries (not shown) were normal in appearance with no endometrioma. Treatment with continuous oral contraceptive pills was initiated after imaging

Deep endometriosis in adolescents is typically diagnosed on MRI. Imaging findings of deep endometriosis on MRI have been well described. On T2W images, deep endometriosis lesions are hypointense compared to muscle. The lesions may be nodular or may manifest as thickening along structures, and the surface of the lesions can be smooth or irregular/stellate. The lesions may or may not be accompanied by hyperintense foci on T1W or T2W images; such foci are thought to correspond to areas of glandular endometrium-like tissue [23]. The retrocervical area (including the torus uterinus, uterosacral ligaments, and posterior vaginal wall) is the most common site of deep endometriosis in adolescents. While no consensus exists, the uterosacral ligaments are probably normal when less than 3 mm in thickness and indeterminate when 3–5 mm in thickness. The ENDOVALIRM group described diagnostic criteria for proximal uterosacral ligament disease (within 2 cm of the torus uterinus), including regular or irregular uterosacral ligament thickening (> 5 mm), nodules along the ligament that are visible in 2 planes, irregular retraction of the ligaments, and hemorrhagic foci along the uterosacral ligaments regardless of the presence of thickening (Fig. 3) [24]. Rectosigmoid deep endometriosis has also been reported at low rates in adolescents [17] This type of deep endometriosis is often contiguous with retrocervical disease and can characteristically cause a thickening of the rectosigmoid wall that has a fan shape or “mushroom cap” appearance.

## Endometriosis and congenital female reproductive tract anomalies

**Fig. 4:**
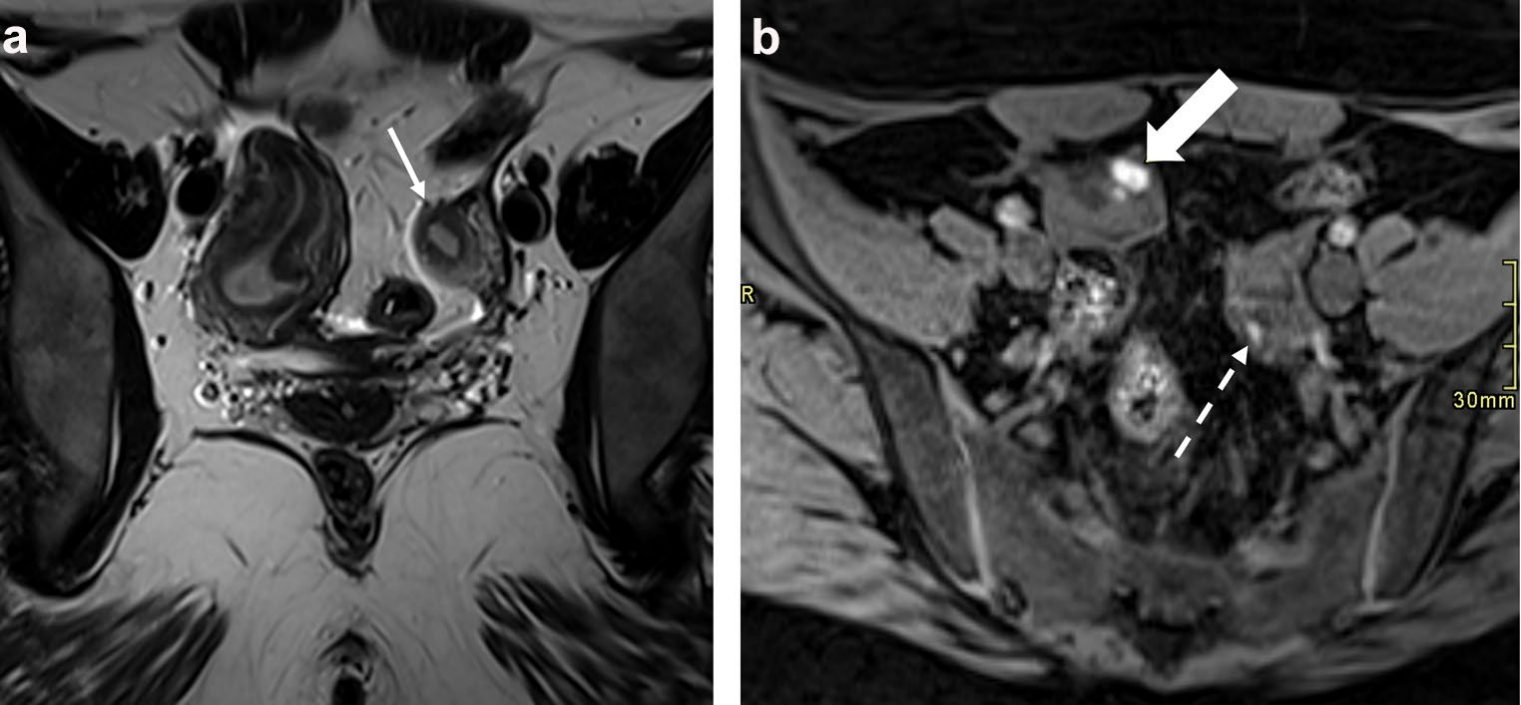
Images from a 22-year-old patient with a known Mullerian anomaly and pelvic pain. **a** Oblique coronal T2W image through the pelvis shows a unicornuate right uterus communicating with the cervix (not shown). A left uterine remnant with functional endometrial tissue (thin arrow) with no communication to the cervix or vagina can also be seen. **b** Axial T1W image shows a right ovarian endometrioma (thick arrow). A T1 hyperintense focus in the left ovary corresponds to a corpus luteum (dashed arrow). Treatment with hormone suppression was initiated. Endometriosis was confirmed on appendectomy

Endometriosis is known to occur in conjunction with congenital female reproductive tract anomalies and has been found in association with all forms of Mullerian anomalies [25]. A recent meta-analysis demonstrated that the rate of endometriosis was significantly higher (47%) among those with obstructive Mullerian anomalies than among those with Mullerian anomalies that were not associated with obstruction (19%) [26]. This study also demonstrated slightly higher rates of endometriosis among those with nonobstructive Mullerian anomalies (23%) than among those without anomalies (21%). The diagnostic appearance and imaging criteria for ovarian endometriomas and deep endometriosis do not differ in the setting of congenital reproductive tract anomalies (Fig. 4). If a pelvic mass or genitourinary malformation is identified, the suspicion for an obstructive Mullerian anomaly and associated endometriosis should be heightened. It is unknown if there are differences in the location of deep endometriosis among individuals with such anomalies.

## Treatment

Treatment strategies for endometriosis include both medical and surgical modalities and often require a multi-modal and multidisciplinary approach.

### Hormonal management

Hormonal suppression with combined hormonal contraceptives or progestins remains the cornerstone of medical therapy for the management of endometriosis and should typically be used as initial therapy for primary dysmenorrhea. Medical management should be considered suppressive and not curative as it will not resolve endometriomas or deep disease but suppresses ovulation and creates a local hypoestrogenic state, which may prevent the development or growth of endometriomas. Of note, although the use of oral contraceptives may improve symptoms, it does not prevent the progression of deep disease or the possibility of future infertility [12]. The treatment choice is dependent on various factors, including lifestyle, comorbidities, tolerability (i.e., ability to swallow pills), and willingness to trial medications. Combined oral contraceptives are often first-line therapy as they are typically well tolerated and accessible. These should be taken in a continuous fashion in an attempt to achieve amenorrhea. Progestin-only options may be preferred when estrogen is contraindicated or there is a desire to avoid unfavorable estrogenic effects. Gonadotrophin-releasing hormone (GnRH) analogues, including agonists and antagonists, may be considered in refractory cases. However, GnRH analogs are associated with bone loss, and adolescence is the time of peak bone accrual. These medications should not be considered without a definitive diagnosis of endometriosis and failure of first-line medication options. If utilized, hormonal add-back therapy with norethindrone acetate and conjugated estrogens should be a part of therapy to preserve bone mineral content [27]. If pain persists after 3–6 months of medical suppression, a diagnostic laparoscopy should be considered for definitive diagnosis and excision [28].

### Surgical intervention

Surgery is indicated when medical management fails or is not tolerated or if the patient desires a definitive diagnosis. The goal of laparoscopic surgery in the adolescent is to confirm the diagnosis and debulk the disease to decrease pain while preserving fertility [8]. Preoperative imaging is crucial for surgical planning and patient counseling. Surgical outcomes are greatly influenced by surgical expertise. Gynecologic surgeons specializing in endometriosis provide optimized outcomes regarding diagnosis, lesion treatment, improved symptom relief, and fertility preservation. Discussion around fertility preservation should be prioritized, especially in patients with bilateral endometriomas, as ovarian reserve can be negatively impacted postoperatively.

A thorough survey of both the pelvis and upper abdomen is crucial to ensure all lesions are identified. Multiple ports are often required to allow appropriate retraction of the uterus and adjacent structures for complete evaluation. Careful attention should be placed on areas found to have potential disease on imaging. Furthermore, when an endometrioma is identified on imaging, there is a > 90% probability that alternate areas of endometriosis are present; therefore, the surgeon should be prepared to address additional areas for excision. Superficial endometriosis lesions in the adolescent population often have a unique phenotypic appearance, including white implants, clear vesicular lesions, or small hemorrhagic areas on the peritoneum, as compared to adults, who often have the characteristic red brown lesions or black, blue, gray “powder burn” lesions. Peritoneal pockets, also known as Allen-Master windows, can be associated with endometriosis and should be everted at the base and excised. Most adolescents are diagnosed laparoscopically with early Stage I or II endometriosis, with advanced Stage III or IV disease being less common [29].

The controversy between laparoscopic ablation versus excision of endometriosis lesions exists because of the paucity of high-quality data in the adolescent population. Excision of lesions is the preferred method for removal and allows for pathologic confirmation, confirmation of complete removal down to the level of normal tissue, and removal over critical structures, including the bowel, bladder, and ureter. If a patient undergoes general anesthesia for a laparoscopy, it is important to consider the option of concurrent levonorgestrel-releasing intrauterine device insertion for post-operative hormone therapy, as intraoperative insertion may negate the need for a painful office procedure for the adolescent patient. Postoperative hormone therapy has been shown to benefit adolescents by decreasing recurrence and slowing disease progression. [30–32].

Adolescents with endometriosis often experience a wide range of symptoms; the frequent overlap with multiple sources of pain, combined with a lack of awareness, can contribute to delays in diagnosis. These pain sources may include gynecologic conditions and non-gynecologic causes, such as gastrointestinal disorders or musculoskeletal issues. This complexity can make diagnosis and management challenging, often necessitating a multi-disciplinary approach. The treatment team may include a pain specialist, mental health professional, physical therapist, and functional medicine specialist [33]. Complementary or alternative treatment options such as acupuncture may assist with chronic pelvic pain. Involving a school counselor or social worker can be beneficial in identifying resources to support classroom engagement and participation in extracurricular activities, as students with endometriosis often face increased absenteeism.

## Conclusion

In the adolescent population, endometriosis has historically been overlooked and underdiagnosed due to atypical presentation and limited awareness among clinicians. When patients are identified, imaging is playing an increasingly important role in diagnosis and surgical planning. While adults may first undergo a transvaginal ultrasound, adolescents have different imaging considerations, with pelvic MRI playing an increasingly important role as it is less invasive and can demonstrate deep endometriosis. Treatment of adolescents is also unique and requires careful thought and consideration of the multitude of effects it may have on quality of life.

## Data Availability

No datasets were generated or analysed during the current study.
